# The role of non-numerical information in the perception of temporal numerosity

**DOI:** 10.3389/fpsyg.2023.1197064

**Published:** 2023-07-31

**Authors:** Guido Marco Cicchini, Giovanni Anobile, David C. Burr, Paolo Marchesini, Roberto Arrighi

**Affiliations:** ^1^Institute of Neuroscience, CNR, Pisa, Italy; ^2^Department of Neuroscience, Psychology, Pharmacology and Child Health, University of Florence, Florence, Italy; ^3^School of Psychology, University of Sydney, Camperdown, NSW, Australia

**Keywords:** numerosity perception, temporal numerosity, approximate number system, visual perception, magnitude perception

## Abstract

Numerosity perception refers to the ability to make rapid but approximate estimates of the quantity of elements in a set (spatial numerosity) or presented sequentially (temporal numerosity). Whether numerosity is directly perceived or indirectly recomputed from non-numerical features is a highly debated issue. In the spatial domain, area and density have been suggested as the main parameters through which numerosity would be recomputed. In the temporal domain, stimuli duration and temporal frequency could be similarly exploited to retrieve numerosity. By adapting a psychophysical technique previously exploited in the spatial domain, we investigated whether temporal visual numerosity is directly perceived. Adult participants observed sequences of visual impulses sampled from a stimulus space spanning several levels of temporal frequency and duration (and hence numerosity), and then reproduced the sequence as accurately as possible via a series of keypresses. Crucially, participants were not asked to reproduce any particular property (such as number of impulses) but were free to choose any available cue (such as total duration, or temporal frequency). The results indicate that while the overall sequence duration was barely considered, numerosity and temporal frequency were both spontaneously used as the main cues to reproduce the sequences, with a slight but significant dominance of numerosity. Overall, the results are in line with previous literature suggesting that numerosity is directly encoded, even for temporal sequences, but a non-numerical feature (temporal frequency) is also used in reproducing sequences.

## 1. Introduction

Numerosity perception, or the “number sense,” refers to the ability that humans share with many animals to make rapid albeit approximate estimates of the number of elements in a set or events in a temporal sequence. In the last few years, much research has been dedicated to numerosity perception as, from an evolutionary point of view, it is of critical importance to make efficient survival decisions such as spotting locations containing large resources of food or making reasonable fight or flight decision depending upon the number of confederates and opponents. As a result of this effort, the knowledge of the functional properties of what is usually refer to as number sense has steadily improved. For example, electrophysiological studies identified neurons with activity tuned to numerosity in the brain of several non-human, animal species such as chicks, fishes, crows and monkeys (Viswanathan and Nieder, 2013; Ditz and Nieder, 2015; Rugani et al., 2015; Nieder, 2016; Agrillo et al., 2017). In line with the ancient nature of this mechanism, behavioral studies in humans revealed that the capacity to perceive spatial and temporal numerosity is already present very early in life (Izard et al., 2009; Coubart et al., 2014; Anobile et al., 2021c), refines during life time (up to 30 years old, see Halberda et al., 2012) and numerosity is a feature encoded spontaneously as indicated by its capacity to modulate the pupillary light response even when observers are not engaged in any explicit task (Castaldi et al., 2021). These results nicely compliment the finding of numerosity-tuned neuronal populations in association areas of the human brain achieved via functional magnetic resonance imaging (fMRI) (Piazza et al., 2004; Harvey et al., 2013; Castaldi et al., 2016; Harvey and Dumoulin, 2017; Paul et al., 2022) and intracranial neural recording (Kutter et al., 2018).

Despite a wide consensus about the brain’s capacity to encode and process numerosity information, there has been a long-standing debate about whether numerosity is sensed directly or derived as a second-order product from the combination of non-numerical features. For example, as perceived numerosity and density are both influenced by stimuli area (with larger arrays being overestimated) some authors suggested that numerosity is the by-product of area and density (Dakin et al., 2011). However, this view has been challenged by studies reporting a spontaneous tendency to focus on numerosity information instead of area or density. For example, Ferrigno et al. (2017) asked participants to learn to categorize sets of dots as representing a “small” or “large” quantity. In the learning phase non-numerical features (area) was positively correlated with numerosity, so participants could use every dimension as the basis of their categorization. Once participants were trained, some probe trials were added. In these trials area and numerosity were uncorrelated to reveal the dimension that the observers spontaneously leveraged on for the categorization. The results clearly showed that numerosity was the driving dimension.

Another paradigm used to reveal the spontaneous focus on spatial numerosity was adapted from color vision (MacAdam, 1942). Cicchini et al. (2016) designed a two-dimensional (2D) stimulus space representing density, area and thus numerosity. On each trial, three stimuli were simultaneously presented, two identical standards and one oddball differing from standards in one of the dimensions described by the stimulus space. Even when not instructed about which dimension they should rely on for the task (numerosity, density or area), participants were more sensitive to numerosity, and spontaneously relied on it to discriminate among the stimuli. Using a different paradigm, closely related to the current study, the spontaneity of spatial numerosity perception was investigated with a free reproduction task. After a brief presentation of a sample cloud of dots, subjects were prompted with a new array which they had to edit (via 2D track-pad movements) to make it as similar as possible to the previous one. No instructions were given about which feature to use to solve the task, with participants being free to match the stimuli for area, density or numerosity. Again, numerosity was the most salient feature, confirming its key role in guiding behavioral responses. Further studies generalized the finding to adolescents (with and without dyscalculia) as well as to preschoolers (Anobile et al., 2019).

It is important to note that all the above studies were about spatial numerosity in which a given number of items are displayed over a given region of space, with all items presented simultaneously. However, the number sense is capable of encoding events numerosity also when these are presented sequentially regardless the stimuli sensory modality being visual (White, 1963; Tonelli et al., 2022), auditory (Arrighi et al., 2014; Grasso et al., 2022), tactile (Togoli et al., 2021; Togoli and Arrighi, 2021) with recent reports indicating that it is also able to encode numerosity of self-produced actions (Anobile et al., 2016; Togoli et al., 2020; Anobile et al., 2021a).

Whether temporal numerosity is sensed directly or indirectly has been investigated much less than spatial numerosity. This is surprising, given that a similar argument can be made about temporal numerosity, whether it is sensed directly or from a combination of temporal frequency and duration. The available studies on this topic were all performed by Stroop-like interference paradigms that do not allow to achieve any definitive conclusions. For example, Agrillo et al. (2010) asked participants to judge the overall duration or the numerosity of tones sequences. Numerosity and sequence duration were pitted against each other with the same duration containing a variable number of tones (and thus a different temporal frequency), and the same number of tones lasting for variable durations. The results on bias did not show any interaction between numerosity and duration (and thus frequency), suggesting independence of the two dimensions. Dormal et al. (2006) asked participants to compare the relative numerosity or duration of visual sequences. The pairs were organized so that in some conditions numerosity and duration were congruent (more numerous lasting longer) or incongruent (more numerous lasting less). The results show that while numerical congruency interfered in the duration task (higher response time for incongruent pairs), the temporal cues did not interfere with visual temporal numerosity, suggesting an asymmetric link between the two dimensions. By leveraging on a similar discrimination task (which sequence contained more flashes), Tokita and Ishiguchi (2011) showed that the number of visual events is underestimated for long sequences (total duration), compared to shorter.

Although they can be informative in demonstrating the automaticity of the encoding of numerosity and/or non-numerical features (such as duration), these interference experiments are not informative about which dimension would dominate perceptual decisions in the absence of a cognitive/perceptual conflict. For this reason, we tackled here this issue directly by adapting the free reproduction paradigm of Cicchini et al. (2016) to investigate spatial numerosity. We created a stimulus space spanning duration, temporal frequency and hence numerosity (the product of the two) and examined which dimensions human observers were most sensitive to when reproducing a sequence of visual events with keypress. Like the studies on spatial numerosity, participants were not instructed to focus on a given perceptual dimension, but just to produce a motor temporal sequence as similar as possible to that previously observed. The results indicate that while numerosity was spontaneously used to reproduce visual sequences, temporal frequency also played a significant role, suggesting a close interplay between these two features in defining the internal representation of quantity for visual events distributed over time.

## 2. Materials and methods

### 2.1. Participants

Eighteen participants, naive to the goals of the study and with normal or corrected-to-normal vision participated in this study (6 men, 12 women, 23–30 years old, mean age 27 years). All participants provided written informed consent.

Experimental procedures were approved by the regional ethics committee (*Comitato Etico Pediatrico Regionale, Azienda Ospedaliero-Universitaria Meyer, Firenze*) and are in line with the declaration of Helsinki.

### 2.2. Apparatus and stimuli

**Figure 1:**
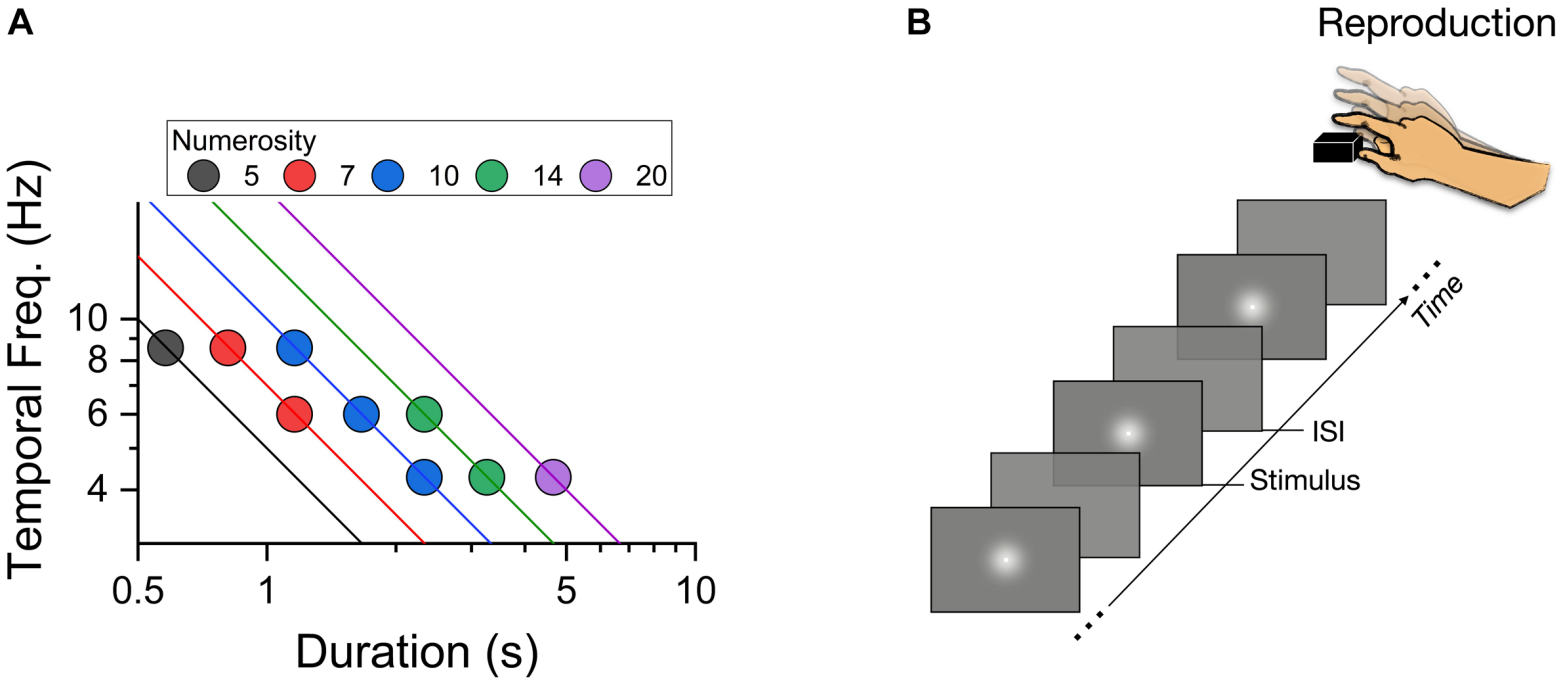
**(A)** Two-dimensional representation of stimulus space, plotting temporal frequency (Hz) of the sequences against total sequence duration (secs). The color-coding of the symbols gives their numerosity, with lines showing constant numerosity on this logarithmic plot. **(B)** Schematic representation of a trial with a sequence of flashes presented at variable ISIs (inter stimulus intervals).

Stimuli were generated and presented via PsychToolbox 3 routines (Brainard, 1997; Pelli, 1997) in The MathWorks Inc. (2022) and displayed on an iMac monitor (1920 × 1,080, 21.5″, 60 Hz). Visual temporal sequences were pre-defined from 9 possible configurations (illustrated in Figure 1A), with total duration (the time elapsed from the first to the last flash) ranging from 0.58 to 4.66 s (0.58, 0.81, 1.16, 1.66, 2.33, 3.26, 4.66), temporal frequencies from 4.28 to 8.57 Hz (4.28, 6, 8.57), and numerosity ranging from 5 to 20 (5, 7, 10, 14, 20). The sequences were created in several steps. First the stimuli (flashes) were assigned to a constant time position, by dividing the total duration by numerosity. Then the temporal distance between successive stimuli was computed and, to avoid regular structures, perturbated by a temporal jitter. The magnitude of the jitter was randomly chosen for each flash and temporal position, following a Gaussian distribution of standard deviation equal to 30% of the average inter-flash separation. All the stimuli employed throughout the experiment were white gaussian blobs with 1.7° Full Width half Maximum displayed centrally for 33 ms each (see Figure 1B). As each flash had a fixed duration, numerosity positively correlated with cumulative flash duration (the sum of flashes duration: N5 = 167 ms, N7 = 233 ms, N10 = 333 ms, N14 = 467 ms, N20 = 667 ms).

### 2.3. Procedure

Participants were sat comfortably in a dimly lit and quiet room, 67 cm from the monitor. The instruction was: “you will now see a sequence of flashes on the screen. At the end of the presentation, you have to reproduce the sequence by a series of key press on the space bar as accurately as possible.” No other instructions or feedback were provided. Each trial started with a central fixation point remaining on the screen for 500 ms. Then a sequence of flashes was centrally presented. At the end of the sequence the fixation point appeared again to signal the start of the reproduction phase with the sequence reproduced by space bar presses (Figure 1B). The sequence presented on each trial was randomly selected between the nine pre-defined combinations of durations, temporal frequencies and numerosity. Each participant completed 120 trials (divided into 4 separate blocks).

### 2.4. Analyses

**Figure 2:**
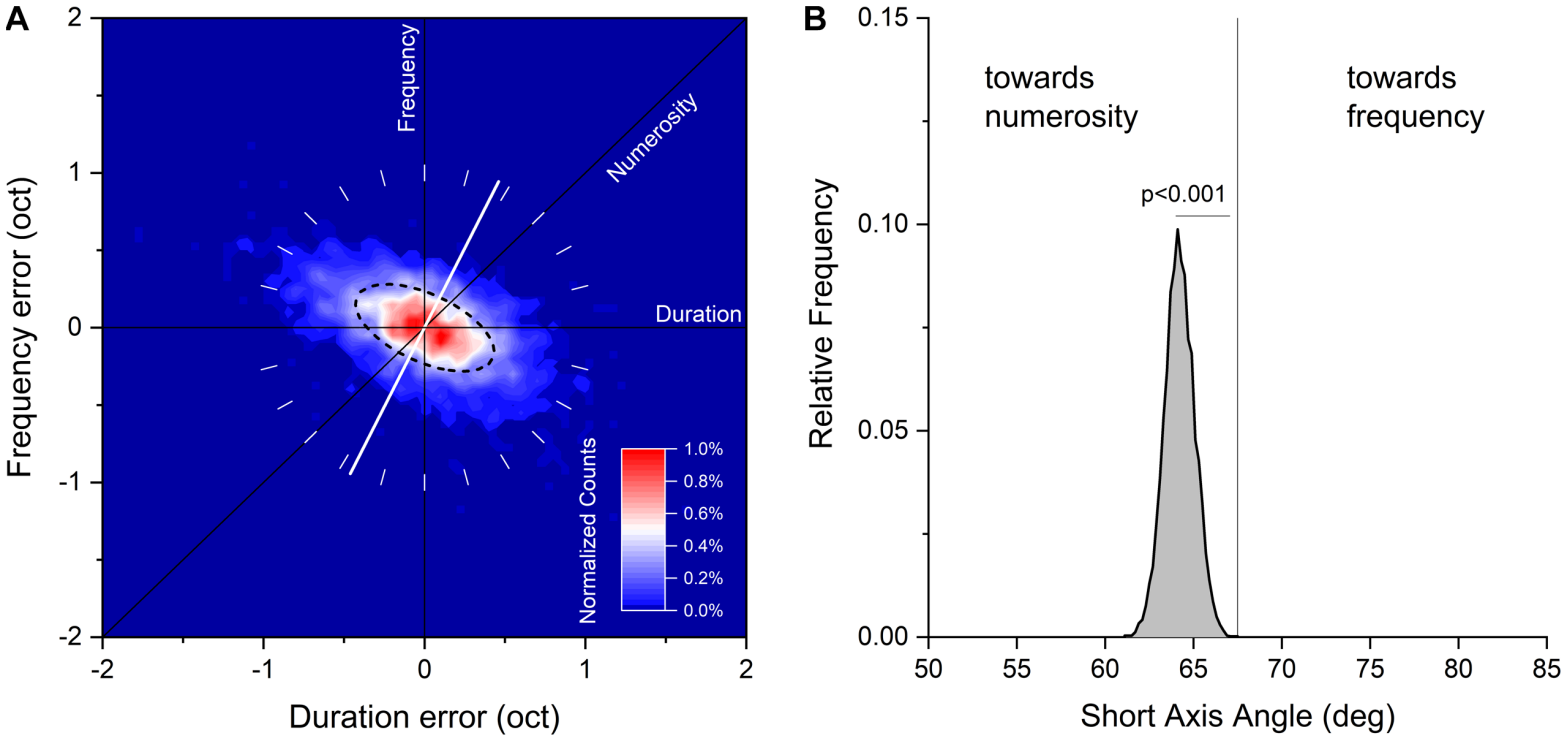
**(A)** Aggregate data showing normalized counts of errors in visual sequences reproduction on logarithmic plot of duration and temporal frequency. Data have been binned every 0.05 octaves and color maps normalized counts in a range from 0 to 1%. The black dashed line corresponds to half height of the fitted Gaussian ellipse fit to the data. The white tilted line runs through the orientation of the short axis of the fit (maximal sensitivity). **(B)** Bootstrap frequency distribution of short axis angle derived from aggregate data a 2D Gaussian fit.

The duration and temporal frequencies of the reproduced sequences of each trial were plotted on a two-dimensional logarithmic space with the abscissa representing the ratio of the reproduced and tested sequence duration, and the ordinate the ratio of the reproduced and tested sequence temporal frequency. The covariance matrix between the two dimensions was then calculated and the eigenvalues and the eigenvectors of the covariance matrix extracted. These correspond to the principal components of the data. From these values, we calculated the best-fitting two-dimensional Gaussian, plotted in the Figure 2A as the ellipse passing though half the maximum of the probability density function. The angle of shorter axis of the ellipse represents the direction of maximal sensitivity, the angle of longer axis the direction of lower sensitivity. It can be easily demonstrated that the hypothetical strategy which blends two sensory features linearly is related nearly linearly to the angle of the ellipse (e.g., if an observer weights 20% sequence duration and 80% numerosity, the short axis of the ellipse will be close to the weighted sum of the two components 0° and 45°, in this case 36°).

The lengths of the axes give an index of sensitivity, with shorter lengths representing higher sensitivities. To quantify length irrespective of the orientation of the ellipse we employed Manhattan distance, which is the sum along the cardinal axis of the graph (hence sequence duration and frequency). Manhattan distance corresponds to total change and can be obtained from the length of the eigenvector (which assumes a Euclidean space) by multiplying it by [sin(α) + cos(α)], where α is the angle of the axis.

The analyses were primarily on the pooled dataset: as different conditions may have different biases, before pooling the various sub-conditions, we removed the average bias of that condition.

## 3. Results

Eighteen adult participants were asked to reproduce irregular sequences of brief flashes varying trial by trial in total duration, temporal frequency and hence numerosity. We first analysed the 9 conditions separately and checked for differences in size and orientation of the ellipses using a one-way Repeated measures Bayesian Anova. Bayes Factors of this analysis were all below 0.4 suggesting that there was no difference between the sub-conditions. All further analysis was therefore on the pooled dataset, after removing the average bias for all participants and conditions.

Figure 2A plots duration and frequency reproduction errors (in octaves), for data pooled across all participants and stimuli configurations. Response distributions were fitted with a 2D Gaussian elliptical function (dashed line): the short axis of the ellipse corresponds to the dimension where sensitivity is maximal, and the long axis the dimension where sensitivity is minimal (for a similar procedure see Cicchini et al., 2016). The angle of the short axis indicates which feature dominates the sequence reproduction: 90° refers to temporal frequency, 45° to numerosity and 0° to duration. From inspection, it is clear that the response distribution lies around the negative diagonal (constant numerosity) with the short axis of the fitted ellipses lying between the numerosity and frequency predictions (64.2° ± 0.85°), indicating that both features were used to reproduce the sequences.

We then asked whether this intermediate behavior was nearer to the numerosity or frequency prediction. If observers weighted numerosity and frequency equally, the predicted axis would be equal to 67.5°: lower if numerosity were weighted more, higher for temporal frequency. To test these predictions, we ran 10,000 iterations of bootstrap (resampling the dataset with replacement, as many indexes as the dataset size), fitted each resample with the 2D Gaussian function and measured the short axis angle. The results are shown in Figure 2B as the bootstrap frequency distribution of the short axis angle together with the “equal numerosity and frequency” prediction (67.5°). The distribution was clearly shifted leftward compared to the “equal numerosity and frequency” prediction, suggesting that although temporal frequency was clearly relevant to the task, numerosity was weighted relatively more (*p* < 0.001, with p reflecting the proportion of trials where the short axis angle was higher than 67.5°).

**Figure 3:**
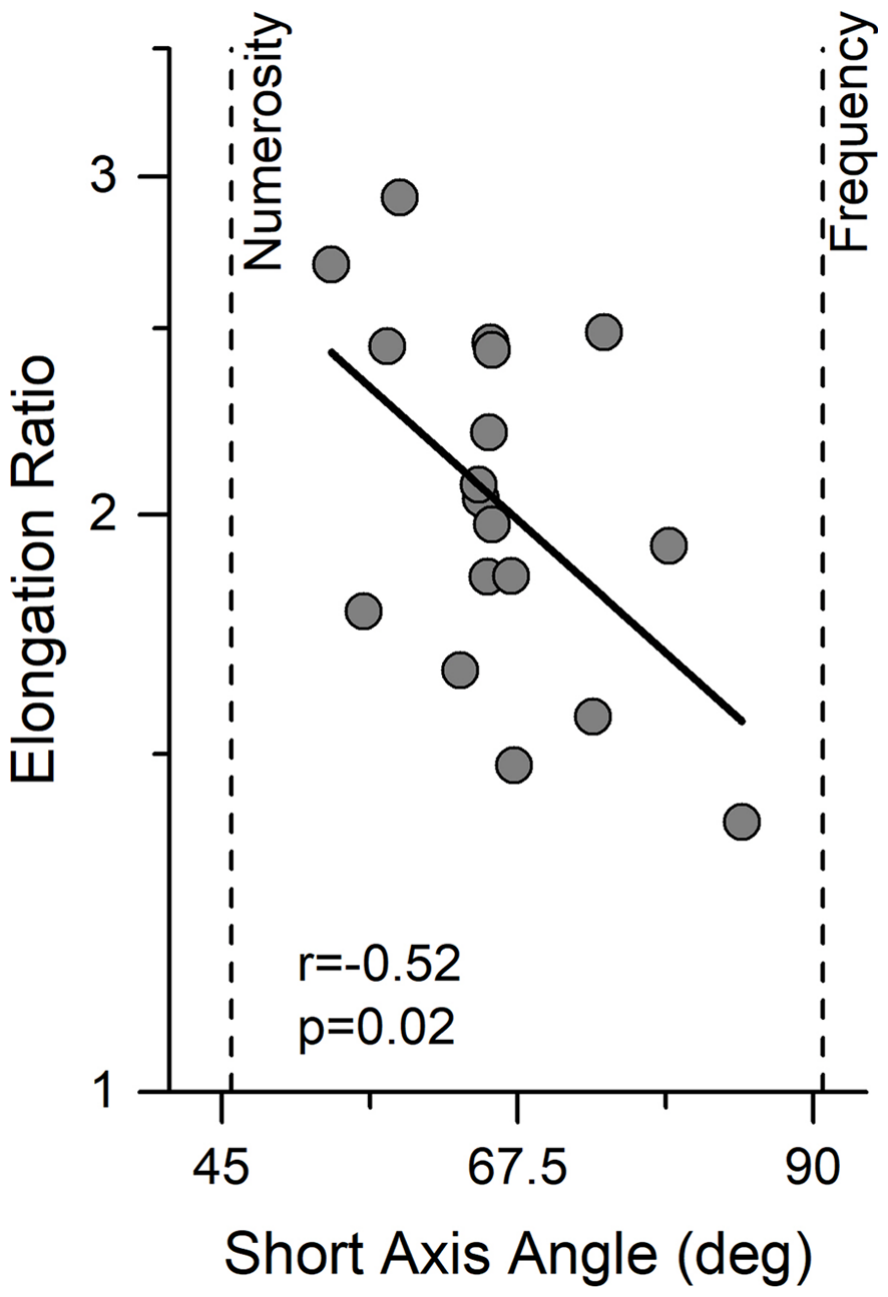
Individual data showing the relationship between angle and elongation ratio. Ellipse elongation plotted against short axis angle. Thick black line indicates linear regression. Vertical lines indicate performance expected by an observer sensitive only to numerosity or temporal frequency.

The same fitting procedure was also applied to single subject data. Figure 3 shows the results for the individual participants, expressed as short axis angle, width of the ellipse (essentially an index of precision), and elongation ratio (ratio of long to short angle length). There are clearly large individual differences in all parameters, with the short axis angle ranging from 55° to 85° (average = 66.1, Sd = 7.8), short axis width ranging from 0.21 to 0.36 oct (average = 0.29, Sd = 0.037) and elongation ratio from 1.3 to 2.9.

Interestingly elongation ratio correlated negatively with the direction of best sensitivity (short axis angle) (r = −0.52, *p* = 0.02) indicating that observers with better performance were those who relied more on the numerosity information.

As a final analysis, we looked at the role of rhythm. As described in the methods section, to avoid the repeated presentation of regular temporal patterns, each flash (within each sequence) was randomly jittered in time, breaking the sequence regularity. A possibility is that, despite not being asked to do so, participants matched the temporal rhythm by preserving the inter stimulus interval (ISI). Although this parameter was not parametrically manipulated in the stimulus space used here, it might still have affected the measured decision angle. To check for this possibility, we calculated regularity of reproduction from the standard deviations of the reproduced ISIs. For each trial we calculated the standard deviation of the reproduced ISIs, and then divided the dataset into two group of trials, having high or low ISIs variability, compared to the dataset median. The datasets were then separately fitted with the 2D gaussian function and the short axis angle extracted. The trials with more regular reproduction produced a decision angle more tilted toward the temporal frequency prediction (68.3° ± 0.99°) compared to the results obtained with the higher median trails that were instead relatively more tilted toward the numerosity prediction (62.1° ± 1.2°).

Similarly, we asked whether stimulus regularity over time could play a role in influencing reproduction strategy. In particular one might expect that stimuli with high regularity to induce observers to use the frequency information more with angles leaning toward 90 degrees. To this aim we limited ourselves to the lowest numerosity, which provided a broad range of regularities across trials (coefficient of variation ranging from 0.15 to 0.35). Again regularity played little role with regular stimuli inducing response distributions oriented at 60.3 ± 9 degrees, and irregular stimuli producing ellipses tilted at 68.7 ± 4 degrees (BF 0.37).

## 4. Discussion

The aim of the current study was to investigate the role of non-numerical cues on temporal numerosity perception of sequences of visual stimuli, with an assumption free task. We measured the cues human participants use when asked to reproduce a sequence of visual events varying in duration, temporal frequency, and numerosity. The results show that participants based perceptual decisions on a mixture of temporal frequency and numerosity, while the overall sequence duration was effectively discarded. Comparing the contribution of temporal frequency and numerosity a slight but significant dominance of numerosity emerges. These data speak against the idea that temporal numerosity is extracted from duration but also highlight the role of temporal frequency as a relevant and perceptually robust non-numerical feature. There was considerable variation between participants, which was interesting. Those who relied more on numerosity than temporal frequency tended to have better precision (more narrow ellipses), as well as more elongated ellipses. This suggests that numerosity is the more useful strategy which, once learned, leads to better performance.

This is not the first investigation of the role of non-numerical cues in the perception of temporal numerosity. However, it is the first study to directly address the use of numerical and non-numerical features by testing this issue eliminating the potential confounds of cognitive strategies driven by explicit task demands specifying the attributes to be judged (Yates et al., 2012). Available studies have generally employed paradigms in which one stimulus feature was explicitly measured (e.g., numerosity) while one or more others features co-varied congruently or incongruently (Dormal et al., 2006; Tokita and Ishiguchi, 2011; Dormal and Pesenti, 2012; Javadi and Aichelburg, 2012). The results showed significant interference between non-numerical temporal information (e.g., stimuli duration) and numerosity perception (but see Agrillo et al., 2010), indicating that both are automatically perceived. The results of the current study are generally in line with this literature, indicating a substantial role of both numerical and non-numerical features, and showing that this interplay emerges even when the paradigm does not require explicit numerical estimations and/or conflicting conditions.

The current results are also in line with the influential ATOM theory (Walsh, 2003) suggesting an integrated system for temporal and numerical information. Even if in the current work we selectively measured temporal numerosity, some parallelism with the investigation of the role of non-numerical cues in the spatial domain might be informative. For example, by leveraging on a spontaneous dot array reproduction task (Cicchini et al., 2019), it has been shown that numerosity largely dominates reproduction behavior over area (the homologous of stimulus duration) and density (the homologous of temporal frequency). The comparison between the two results suggests that spatial numerosity might be more robust to the influence of non-numerical cues, compared to temporal numerosity.

It should be mentioned however that the reproduction tasks employed to test spatial and temporal numerosity are similar but not identical. While for spatial numerosity participants were asked to edit a sample dot-image provided by the software, in the case of temporal numerosity participants had to freely reproduce the temporal sequence. To avoid the repeated presentation of regular temporal patterns, the individual events (flashes) were interspersed with irregular pauses (see methods for details). These procedures might had masked the spontaneous use of duration, temporal frequency or numerosity, triggering the reproduction of the internal temporal structure (rhythm). While the stimulus space used here did not parametrically manipulate this cue, some hints exist. Trials in which the reproduction was more regular in time had a decision angle more tilted toward the temporal frequency prediction compared to the results obtained time-irregular trails. These results suggests that those participants more spontaneously tuned to numerosity tended to produce more irregular patterns, probably spontaneously employing chunking strategies, a behavior that has been proved to be used in the perception of spatial numerosity (Anobile et al., 2020, 2021b, 2022; Maldonado Moscoso et al., 2022).

A possible artifact may be that that participants were more tuned to the feature free to vary more within the stimulus space, as has been suggested for spatial numerosity (Salti, 2022). However, that would appear not to be the case, as the feature varying most was total duration, which varied by a factor of 8 (from 0.58 to 4.66 s), while temporal frequency varied by only a factor of 2 (from 4.28 to 8.57), and numerosity by a factor of 4 (from 5 to 20). In brief, the stimulus dimension that varied the most (duration between the first and last flash) was the least used by participants in reproduction (the less behaviorally salient).

On a similar note, our results demonstrated that stimulus regularity played little role in the reproduction strategy. In our experiment stimulus regularity was not manipulated parametrically but did vary because of the intrinsic variability in the sequence generation algorithm. Comparing the more regular with the more irregular stimulus sequences revealed no difference in reproduction precision. It would be interesting to extend this finding in future studies by checking if, even in the case of extremely regular intervals, observers would still represent and reproduce sequences by taking into account numerosity.

Another aspect emerging from the current results is the high individual variability in the angle of max sensitivity. Although many participants showed highest sensitivity angle around 65° (a near perfect mixture of numerosity and temporal frequency), the individual data spanned from around 50° (near numerosity prediction) to around 85° (near duration prediction). Interestingly, the angle of maximal sensitivity positively correlated with the elongation of the ellipse of the short axis, an index of sensory precision. This result suggests that temporal numerosity perception (at least under conditions of free reproduction) is a complex process that is likely to integrate information from multiple noise sources with the weights assigned to the different cues varying robustly between observers. In other words, the behavioral response (the elongation ratio in this case) could reflect the relative weights assigned to the various sources of information. In this specific case, a subject who uses almost exclusively numerosity will produce sharper dispersion distributions than observers who use a mixture (or a pool) of strategies. Finally, the current results, while suggesting a stronger role of non-numerical cues on temporal relatively to spatial numerosity perception, do not stand against the previously suggested idea for the exitance of an integrated system encoding both (Arrighi et al., 2014; Anobile et al., 2021a). Indeed, while the relative weight of non-numerical cues on temporal and spatial numerosity might be different, in both cases numerosity turned out to be a salient feature (that driving more perceptual decisions). The numerical information conveyed by both temporal and spatial numerosity could thus represent the basis of the behavioral results showing a clear sensory crosstalk between the two domains, as the case of cross-format numerosity adaptation (Arrighi et al., 2014).

The current study is the first measuring the influence of non-numerical cues on temporal numerosity perception, minimizing all possible task-driven confounds. The results highlight the possibility that temporal frequency is involved in the numerical process. The task used is relatively fast, simple, intuitive, and flexible, making it suitable to further parametric manipulations and for its use in early developmental stages. This study may open to new technique to quantitatively and ecologically investigate how the relative weights spontaneously assigned to different stimulus features (also in non-visual modalities) shape numerical encoding, also during development.

## Data availability statement

The original contributions presented in the study are included in the article/supplementary material, further inquiries can be directed to the corresponding author.

## Ethics statement

The studies involving human participants were reviewed and approved by Experimental procedures were approved by the regional ethics committee (Comitato Etico Pediatrico Regionale, Azienda Ospedaliero-Universitaria Meyer, Firenze) and are in line with the declaration of Helsinki. The patients/participants provided their written informed consent to participate in this study.

## Author contributions

DB, GC, GA, and RA developed the study concept and contributed to the study design. PM performed the testing and data collection. DB and GC performed data analysis. GC and GA drew the figures. All authors contributed to data interpretation and drafted the manuscript. DB provided critical revisions. All authors contributed to the article and approved the submitted version.

## Funding

This research was funded by the European Union (EU) and Horizon 2020—Grant Agreement no. 832813—ERC Advanced “Spatio-temporal mechanisms of generative perception — GenPercept”; and from the Italian Ministry of Education, University, and Research under the PRIN2017 program (Grant no. 2017XBJN4F— “EnvironMag”).

## Conflict of interest

The authors declare that the research was conducted in the absence of any commercial or financial relationships that could be construed as a potential conflict of interest.

## Publisher’s note

All claims expressed in this article are solely those of the authors and do not necessarily represent those of their affiliated organizations, or those of the publisher, the editors and the reviewers. Any product that may be evaluated in this article, or claim that may be made by its manufacturer, is not guaranteed or endorsed by the publisher.
